# A transformative mission for prioritising nature in Australian cities

**DOI:** 10.1007/s13280-022-01725-z

**Published:** 2022-03-29

**Authors:** Niki Frantzeskaki, Cathy Oke, Guy Barnett, Sarah Bekessy, Judy Bush, James Fitzsimons, Maria Ignatieva, Dave Kendal, Jonathan Kingsley, Laura Mumaw, Alessandro Ossola

**Affiliations:** 1 Department of Human Geography and Spatial Planning, Faculty of Geosciences, Utrecht University, Utrecht, The Netherlands; 2 Centre for Urban Transitions, Swinburne University of Technology, Melbourne, Australia; 3 Connected Cities Lab, Faculty of Architecture, Building and Planning, The University of Melbourne, Melbourne, VIC 3010 Australia; 4 CSIRO Land and Water, Clunies Ross Street, Canberra, ACT 2601 Australia; 5 ICON Science, RMIT University, Melbourne, VIC 3001 Australia; 6 Faculty of Architecture, Building and Planning, The University of Melbourne, Melbourne, Australia; 7 The Nature Conservancy, Suite 2-01, 60 Leicester Street, Carlton, VIC 3053 Australia; 8 School of Life and Environmental Sciences, Deakin University, 221 Burwood Highway, Burwood, VIC 3125 Australia; 9 School of Design, the University of Western Australia, M433, Perth, WA 6001 Australia; 10 Healthy Landscapes Research Group, School of Geography, Planning and Spatial Sciences, University of Tasmania, Hobart, TAS 7000 Australia; 11 School of Health Sciences, Swinburne University of Technology, 12 Wakefield Street (Swinburne Place West), Melbourne, VIC 3122 Australia; 12 Centre for Urban Research, RMIT University, 124 La Trobe St, Melbourne, VIC 3000 Australia; 13 Gardens for Wildlife Victoria, 511 Burwood Hwy, Wantirna South, VIC 3152 Australia; 14 Department of Plant Sciences, University of California, Davis, CA USA; 15 Department of Biological Sciences, Macquarie University, North Ryde, NSW Australia; 16 School of Ecosystem and Forest Science, The University of Melbourne, Burnley, VIC Australia

**Keywords:** Indigenous knowledge, Metropolitan, Nature-based solutions, Planning, Policy, Urban

## Abstract

Australia is experiencing mounting pressures related to processes of urbanisation, biodiversity loss and climate change felt at large in cities. At the same time, it is cities that can take the leading role in pioneering approaches and solutions to respond to those coupling emergencies. In this perspective piece we respond to the following question: *What are the required transformations for prioritising, valuing, maintaining and embracing nature in cities in Australia?* We adopt the mission framework as an organising framework to present proposed pathways to transform Australian cities as nature-positive places of the future. We propose three interconnected pathways as starting actions to steer urban planning, policy and governance in Australian cities: First, cities need to establish evidence-based planning for nature in cities and mainstream new planning tools that safeguard and foreground urban nature. Second, collaborative planning needs to become a standard practice in cities and inclusive governance for nature in cities needs to prioritise Aboriginal knowledge systems and practices as well as look beyond what local governments can do. Third, for progressing to nature-positive cities, it is paramount to empower communities to innovate with nature across Australian cities. Whilst we focus on Australian cities, the lessons and pathways are broadly applicably globally and can inspire science-policy debates for the post COP15 biodiversity and COP26 climate change implementation processes.

## Introduction

Cities are interlinked systems of systems; they consist of ecosystems, economies, cultures, politics manifesting through different visions of the future but ultimately, they are places for people and nature (Gehl 2010). Over recent years knowledge is gathered—theoretical and experiential—on the importance of safeguarding nature in cities to strengthen the resilience of people and the planet to climate change, biodiversity extinction and environmental degradation (UNEP 2021). Cities have the governance capacities to respond to these crises (Oke et al. 2021). A variety of voices and institutions shepherd public (and private) green spaces and urban ecosystems through policies, strategic plans and programmes, in systems of constant flux, where the role of, or, location for nature in cities is contested (Dorst et al. 2019). Leaving nature in place, or creating new spaces for nature in cities demands the transformation of planning approaches, policy mechanisms and community practices to put nature at the forefront (Shade et al. 2020; Ossola and Lin 2021). A social, ecological and economic case can be made for prioritising nature-based solutions in Australian cities (Lin et al. 2021). Examples of nature-based solutions include water sensitive and biodiversity sensitive urban design (Garrrand et al. 2018; Kirk et al. 2021) rather than traditional built infrastructure and engineering solutions (Allan et al. 2020; Coutts et al. 2013; Kabisch et al. 2017; Ignatieva et al. 2018, 2020; Frantzeskaki et al. 2019; Keeler et al. 2019).

A macro-driver affecting urban landscapes in Australia is the pace of urbanisation. Australia is a nation of cities and towns. We use the term Australian ‘cities’ to indicate metropolitan areas rather than jurisdictional boundaries, as most Australian cities are governed by multiple local governments as well as state governments. We would like to respectfully acknowledge that Australia’s cities are built on the lands of Aboriginal First Nations peoples whose relationship with both lands and waters through connection to and caring for Country is ongoing and has existed for tens of thousands of years (Miller 2021). In 2016, almost 80% of Australia’s Aboriginal peoples lived in urban areas. Australian cities continue to grow rapidly, having one of the fastest urbanisation rates in (what it is considered) the western world—89% of the population living in a handful of urban areas. Urban development has been preferenced over nature, with economic pressures and imperatives competing with the need to protect natural landscapes (Champness et al. 2019).

Another macro-level driver of change is climate change. Australia as a continent faces pressures such as more frequent droughts, bushfires and extreme heat events that are affecting the resilience of city infrastructures and city dwellers alike now and in the future (Boer et al. 2020). Many climate change pressures are magnified for Australian cities, (Norman et al. 2021), for example most of Australian cities are coastal and face increasing pressures from sea-level rise (Threlfall et al. 2021). The liveability and character of Australian cities is in jeopardy if “climate-ready” interventions and policies are not promptly implemented (Ossola and Lin, 2021).

Against this context, it is recognised that if Australian cities are to contribute in achieving the global Sustainable Development Goals (SDGs) and strive for livability and resilience, transformations in ways of planning, governing and relating with nature are required (Australian Academy of Science 2021). The exploratory research question that guides us in this perspective piece is: *What are the required transformations for prioritising, valuing, maintaining and embracing nature in cities in Australia?* We adopt the mission framework of Mazzucato (2018) as an organising framework to present proposed pathways to transform Australian cities as nature-positive places of the future. The mission-oriented framework has its origin in innovation economics and proposes linking policy and strategy formulation for transformative actions to mission statements that fundamentally and radically progress sustainability challenges (Heikkinen et al. 2019; Sachs et al. 2019). The mission-oriented framework can help to mobilise ideas and develop proposals for prioritising planning of urban nature to enact sustainability transitions in cities.

The paper is organised as follows: In Sect. 2 we present a concept of a transformative mission for prioritising, valuing, maintaining and embracing nature in Australian cities. We present three transition pathways that are interconnected and collectively can enable the required transformations. We elaborate on the challenges, the existing good practices as well as the knowledge bases that can support and activate these pathways in Australian cities. We conclude with Sect. 3 by linking our proposed mission-oriented approach for nature in Australian cities to a globally relevant agenda by highlighting key messages and implications aligned with COP15 of the Convention on Biological Diversity in Kumming, China, the United Nations Convention Climate Change (UNFCCC) COP 26 meeting in Glasgow, UK and Intergovernmental Panel on Climate Change review cycles (AR6-AR7).

## A transformative mission for nature in Australian cities

### A transformative vision for nature-positive cities

The visions and narratives on urban nature in Australian cities need decisive shifting (Australian Academy of Science 2021) to put nature front and centre in all new efforts related to urbanisation, renewal and densification. Whilst often relegated to a lesser urban land use, urban nature needs its urban locus—the physical and metaphysical place where it can grow and thrive. Existing ‘urban futures’ paradigms remain human-centric, employing greening strategies at the margins of urban planning and doing little to confront the drivers of urban development (Daniels et al. 2020). Alternative visions for securing social wellbeing benefits through fostering sustainable human-nature relationships are rarely considered (Ison and Straw 2020) due to short-term governance approaches with limited civil society participation (Mercon et al. 2019; Clark and Harley 2020). Recent scholarship points to the notion of nature-positive economy and futures as an attainable and desirable state for the planet in order to restore planetary boundaries and live in a safe and equitable operating space (Raworth 2017). The focus of nature-positive economies and futures is specifically advocated by World Economic Forum (WEF), stating that a nature-positive future is a goal to work for the coming decade and “a nature positive approaches enhances the resilience of our planet and our societies) (WEF 2020). According to WEF (2020, p. 59) “a nature-positive built environment shares space with nature, putting whole ecosystems rather than humans alone at the centre of design. (…)”. Stemming from this proposal of nature-positive urban environment, we propose to look closer to how nature-positive cities (or even broader, a nature-positive urbanism) can be realised. Specifically, we propose that nature-positive development /future needs to be the aim for our urban systems as well.

By shifting to a narrative of ‘nature-positive cities’, nature can be valued and governed as a formal urban asset with equal scope, worth and importance as traditional capital and infrastructural assets (Buse et al. 2018; Matsler 2019). Urban planning and design need to allow nature to permeate urban landscapes by removing physical and planning barriers that hamper or limit nature integration, to encourage a nature-positive urbanism in Australian cities. In doing so, cities can ensure that urban nature is not only retained but that positive outcomes can boost its performance, resilience and adaptability to old and new global and climate challenges (Davidson and Gleeson 2018; Daniels et al. 2020; Hobbie and Grimm 2020; Ossola and Lin 2021), and the plethora of its benefits for cities, (McPhearson et al. 2016; Kabisch et al. 2017; Dumitru et al. 2020; Padma et al. 2020; Almenaar et al. 2021), noting an acknowledged bias of research into larger cities, and the need for more knowledge of benefits of urban nature, and associated environmental justice issues, for smaller to medium cities (Kendal et al. 2020).

Whilst cities are diverse and complex, they offer plentiful opportunities to plan, retrofit, enhance, design urban nature at different scales, from city masterplan to urban neighbourhood, private yard, public park or verge garden (Ignatieva 2010; Frantzeskaki et al. 2020; Kingsley et al. 2021a). Many facets of urban nature are adaptable, scalable and portable. That will require a shift of knowledge paradigm and practice of landscape architecture, to a biodiversity driven and benefiting landscape architecture approach as an alternative to the existing global homogenised picturesque-gardenesque approach (Ignatieva 2018; Alexandra and Norman 2020).

Nature-positive cities will be planned and managed differently to reflect and support the character and species of nature that belongs to them. The cultivated ‘beautified’ nature of exotic flower gardens, uniform avenues of street trees and carefully managed turf grass may still be favoured, but indigenous wildlife (plants and animals), complex native habitat structure, large trees with hollows and broken limbs, would all be valued and integrated into the landscape. Critically endangered remnant native grasslands would not be destroyed but would be an inspiration for public parks and gardens (DELWP 2016), nor, for example, would grey-headed flying fox camps be dispersed (Currey et al. 2018). Decisions on managing wildlife, or reintegrating nature into cities, need to go beyond human-centric concerns and consider environmental and biodiversity harms and benefits (Maller 2021). Truly nature-positive cities would allow all forms of nature in, to be experienced by urban dwellers (Robertson 2018; Kolokotsa et al. 2020; Mata et al. 2020). This has the potential to benefit humans and nature by mobilising an integrated ethic and practice of caring for human and ecological communities, progressing the possibility of taking on board Indigenous knowledge systems and practices like outlined in Caring for Country (Woodward et al. 2020).

We propose three interconnected pathways as starting actions to steer urban planning and urban policy and governance in Australian cities towards nature-positive urban futures:

- Establish evidence-based planning for nature in cities;
- Strengthen collaborative planning and inclusive governance for nature in cities; and,
- Empower communities to innovate with nature.

### Pathway 1: Establish evidence-based planning for nature in cities

A first pathway to drive urban agendas and actions towards nature-positive cities is to establish evidence-based metropolitan and urban planning for valuing, prioritising and maintaining nature in cities. Urban planning has the potential to bridge the gap between aspirations and real-life transformations in urban spaces and infrastructures, especially when informed and guided by evidence through data, accounting for lived experiences of people and a new appreciation of people-nature relationships in cities (Bush 2020; Potter 2020; Voskamp et al. 2021). Urban planning as an institutional platform to enable and accelerate the transition to nature-positive urbanism can elicit this by transforming planning instruments and approaches, including how urban parks are managed and regenerated, how offsetting is regulated, how zoning and urban development is managed and how new planning tools are integrated in existing urban planning processes.

Urban parks and open space are often important places for nature and for residents to experience nature. These spaces have been significant for city residents during COVID-19 lockdowns in many parts of the world, as places of exercise and contact with nature (Hockings et al. 2020; Ugolini et al. 2020). However, their governance and management are often fragmented, creating challenges for planning connectivity between parks and permeability of a city for nature (Ignatieva et al. 2008; Maron et al. 2016; Tzoulas et al. 2021). Greater inventory and coordinated management between councils, parks agencies, water authorities, planners, designers and developers is required to ensure these areas are managed as a network for people and nature (Sharifi et al. 2021a, b). Metropolitan-wide visioning, strategies and planning are important since they enable more holistic consideration of needs across the landscape-scale at which many species function (such as *Living Melbourne: Our Metropolitan Urban Forest*; The Nature Conservancy and Resilient Melbourne 2019; Hartigan et al. 2021).

Urban development is a major threatening process for nature within and on the fringes of cities. Whilst there may be legislation to protect threatened or vulnerable species and ecosystems within cities, biodiversity offsetting schemes (where biodiversity losses in one place are ‘offset’ in another place) often result in questionable ecological outcomes (Bull et al. 2013; Maron et al. 2016). Reforming offsetting metrics and assessments in the urban landscape is important given that offsetting often does not account for the ‘place-based’ value of nature and results in a localised loss of biodiversity in the places where people live (Garrard et al. 2018; Kalliolevo et al. 2021).

Remnant natural areas in cities are often critical for flora and fauna alike. Both large and small patches can be significant locations for biodiversity conservation with large remnants shown to contain a greater variety of species for some fauna groups (Palmer et al. 2008; Fitzsimons et al. 2011) and small patches often containing the last representatives of the most heavily impacted species and ecosystems (Wintle et al. 2018). Maximising retention of remnant areas when planning newly developing suburbs as well as restoration of natural areas on unused open space or sites decommissioned from other uses have significant positive impacts for nature and needs to be incentivised (Allison 2018). For these processes, it is important for urban planning to consider ecological and biodiversity evidence, research and monitoring, as well as to tap into interdisciplinary knowledge of planners and ecologists (White et al. 2005; Bohnet 2010; Williams et al. 2021). This is specifically relevant for small and medium size cities in Australia located in proximity to remnant natural areas and ecosystem reserves that post-pandemic may seem as desirable areas for peri-urban development. Another aspect to consider in small and medium size cities planning for urban nature is how urban form and biodiversity in urban green spaces relate and in result affect human and ecosystem health (Kendal et al. 2020, p. 6).

Planning schemes and instruments need to better protect features important to nature in cities (e.g. large trees, hollows), with many trees on private land lacking any mechanisms to ensure or promote their retention (Ordóñez-Barona et al. 2021; Wolf et al. 2020; Clark et al. 2020). Urban planning needs to consider lessons and mechanisms from efforts to protect nature on private land in rural landscapes, whilst accommodating the different social and ecological features of cities (Fitzsimons and Wescott 2001; Prado et al. 2018). New urban planning tools need to be integrated into existing planning practices and processes, for example, to connect data on tree canopy cover and other environmental indicators and outcomes to future planning decisions such as the Green Factor Tool (see Box 1).

BOX 1: Green factor tool, city of Melbourne We need to foreground and require green in new developments with tools such as City of Melbourne’s Green Factor which specifies minimum amounts of greening—in-ground, green roofs, walls or facades, in new developments—whether in residential, commercial or industrial settings (Bush et al. 2021). Drawing on experience and research from global examples of integrating greening into urban planning processes, such as Berlin’s Biotope Area Factor, and Seattle’s, Helsinki’s and Malmo’s Green Factor Tools (Bush et al. 2021), City of Melbourne developed the Green Factor Tool to quantify the ratio of greening to built form in new developments. The tool, the first of its kind in Australia, brings together City of Melbourne’s environmental policy objectives (including urban heat mitigation and biodiversity habitat provision), with research on how different forms of vegetation (trees, shrubs, ground cover) can deliver a range of ecosystem services (Bush et al. 2021). The tool allocates weighted points to different forms of greening, and importantly, assigns additional points for inclusion of a maintenance plan, in an attempt to influence the post-development establishment success for greening. Indeed, a key critique of development-based green factor ratings is the lack of integrated monitoring and enforcement efforts (Juhola, 2018). Just as we have a building code, we need a greening code that sets minimum standards, is enforceable and enforced. We also need incentives to reward going beyond minimum standards and demonstrating best practice, including voluntary certification approaches (such as developed by the Green Building Council of Australia) (Bush 2020).

### Pathway 2: Strengthen collaborative planning and inclusive governance for nature in cities

The need for strong environmental policy at state and federal levels needs to go hand in hand with good governance and good planning by local government to support the prioritisation, design, implementation and maintenance of nature in cities. Australian cities operate under policy and planning cycles of state and federal governance levels that are not always synchronised with the needs and pressure at the local level. Despite this, there have been some bold metropolitan and local governance experiments, with Melbourne’s *Living Melbourne* Urban Forest strategy leading the way (Fasternrath and Coenen 2021; Moloney and Doyon 2021). To progress mainstreaming of nature-based solutions in Australian cities, Melbourne’s experience and recently City of Sydney’s Urban Forest Strategy point to the importance of collaborative planning approaches and cross sectoral partnerships from formulation to implementation. New forms of collaboration need not only to be experimented with as ways to innovate urban planning (Bush 2020) but also as ways to adapt urban planning institutions and processes to co-create and co-produce strategy formulation and science-to-policy translation (Coenen et al. 2020). In this it is critical to adopt a cross-sectoral approach that bridges interests and brings together different forms of knowledge (Malekpour et al. 2021), that specifically prioritise Indigenous knowledges and knowledge systems, alongside input from groups often shut out of access to nature due to a range of accessibility issues (Levinger et al. 2021).

Aboriginal communities living in urban areas can be supported to maintain nature-based cultural practices. A critically important practice is cultural burning, and fire is needed to maintain the ecological of many Australian ecosystems such as grasslands and heathlands—the absence of fire can lead to significant ecosystem decline. Reintroducing cultural burning to natural ecosystems in urban landscapes (where it can be done safely) can support culture and ecosystem health (Darug Ngurra et al. 2019). Overall, a more comprehensive recognition and representation of Aboriginal perspectives and knowledge (e.g. Caring for Country principles, Woodward et al. 2020) could benefit urban policies by informing sustainable practices in shaping the environment, reducing disadvantage and strengthening the cultural heritage of Australia’s cities (Kingsley et al. 2013, 2021b; Markevych et al. 2017; Porter 2020; Terare et al. 2020; Egerer et al. 2021; Mumaw and Mata 2021). Building from recent developments on how to approach decolonisation of knowledge and planning systems (Nagendra et al. 2018; Wright 2018; Ludwig and Macnaghten 2020; Trisos et al. 2021), and embracing the Caring for Country principles, Australian cities can find ways to decolonise nature-positive cities.

When it comes to nature, what happens on private or public land affects whole ecosystems and communities. Inclusive governance approaches need to also consider and designed to empower communities, particularly civil actors, to be decision makers and carers for nature (Ison and Straw 2020), including private citizens fostering nature on their property to complement government activities (Mumaw and Mata in press). Planning and governing in an equitable, open and therefore inclusive way ensures that all interests, perceptions, expectations and needs are heard and considered (Cohen-Strachan et al. 2019; Fors et al. 2021). At the same time, inclusive governance need to consider ways to be inclusive to ‘more-than-humans’ and give voice and agency to nature (Apfelbeck et al. 2020; Maller 2021; Pineda Pinto et al. 2021). New forms of inclusive governance can build upon and extend existing good practices such as the *Yarra River Protection (Wilip-gin Birrarung murron) Act 2017* and Birrarung Council in Melbourne (see Box 2) as well as the Managing with Fire programme in the city of Adelaide or the Chain of Ponds Initiative for Moonee Ponds Creek in Melbourne.

BOX 2: Giving a voice to the river: the Yarra River Protection (Wilip-gin Birrarung murron) Act 2017 and Birrarung Council Nature’s multifunctionality is widely recognised and celebrated, from biodiversity habitat, to cooling cities, treating water and air and providing space for social connections and mental and physical health and wellbeing. We need to shift our current monofunctional governance and management arrangements, to develop new approaches that can both accommodate nature’s multifunctionality as well as actually make the most of this multifunctionality (Bush 2020). Participatory approaches are one of these mechanisms. An example is the Victorian *Yarra River Protection (Wilip-gin Birrarung murron) Act 2017*, which aims to give a voice to nature through the establishment of the Birrarung Council statutory body, with representatives from Traditional Owners, environmental and agricultural industry groups, local community groups (O’Bryan 2019).

### Pathway 3: Empower communities to innovate with nature

Urban communities are diverse, and supporting different communities’ innovative practices with nature is an important pathway to inclusive and diverse nature-positive cities that have broad public support and engagement. Importantly, nature is place-based and so are people’s relationships to those places within cities and the biodiversity that constitutes them (Fish et al. 2016; Mattijssen et al. 2020). It is important to ensure that communities of practice that act on protecting, valuing, stewarding nature are not only recognised but further empowered to innovate with nature (Frantzeskaki et al. 2019). Increasingly, professional urban nature-based communities of practice such as arborists, horticulturalists and landscape architects, as well as ecological restoration practitioners, conservationists and residents are innovating to create nature-positive cities. Urban areas allow techniques such as green roofs (Fleck et al. 2022; Wooster et al. 2022), wildlife gardening (Mumaw and Mata in press), chainsaw tree-hollows, ‘planting’ native mistletoe on non-native Plane Trees and detailed modelling of expanded or under-threat habitat areas in cities to be used to support a myriad of urban natures (Griffiths et al. 2018) and renature spaces in the cities.

One advantage of the historically low-density of many Australian cities is that it provides the opportunities for embracing nature and the possibilities of its stewardship across private and public land, such as urban gardening and urban agriculture (Kingsley et al. 2021a). Gardening has traditionally been one of the most popular leisure time activities in Australia, and renewed interest in different forms of gardening (indoor plants, balcony gardening, a renaissance in vegetable gardening) is creating new opportunities for all urban Australians to directly participate in nature-positive activities. Additionally, wildlife gardening has furthered the connection of urban residents with nature: it can function as communities of practice in which residents wildlife garden to support nature stewardship work on public land in partnership with their local governments (Mumaw and Raymond 2021). Learning by doing, involvement of council and community and communication hubs for reminders, motivation and celebration sustain these communities of practice (Mumaw 2017). Wildlife gardening done in partnership between local government and community can provide benefits for biodiversity, personal wellbeing and community capacity building, offering a model for other urban biodiversity conservation initiatives (Mumaw et al. 2019). Such approaches however need to be tailored to the city-specific context taking into account the diversity of small to big cities since people-nature connections are mediated and understood differently across Australian cities (Kendal et al. 2020).

Overall, these diverse communities can be supported by removing regulations that prevent innovation, e.g. on nature-strip (or verge) gardening (Marshall et al. 2020), as well as encourage and province explicit support for culturally and socioeconomically diverse communities to participate in nature-positive practices, such as community gardens, gardening for wildlife programmes and foraging (Oke et al. 2021) (see Box 3).

BOX 3: Gardens for Wildlife Victoria In 2016, Gardens for Wildlife Victoria was launched to develop a network of champions from community and government to mentor and learn from each other to codesign and deliver wildlife gardening programmes (Gardens for Wildlife Victoria 2021). The network showcases and promotes principles shown to foster success in wildlife gardening programmes (Mumaw and Bekessy 2017). A core principle is forming community-council partnerships in each local government area—community partners may be volunteer groups or individuals—to develop and run locally sited programs, designed by locals and suited to local aspirations, strengths and species. Another is to target social alongside environmental goals, for example to involve recent immigrants or assist in bushfire recovery. From 2016, the network has spread from 1 to 41 of the state’s 79 local government areas, from inner city to semi-rural townships and from one program to 16 operational, 14 developing and 11 programs in early exploration. The network has over 300 members. Program developers and leaders recount four key themes for what empowers them to persist in their program development activities, themes claimed to underpin empowerment in work-related scenarios (Thomas and Velthouse 1990): having impact, doing something meaningful to you, having the capability to do it and having a choice (Mumaw and Raymond 2021). Examples of working models, trusted relationships, a safe space to think differently and sharing of successes and challenges were key resources provided by the network that stimulated sustained innovation in program development and delivery (Mumaw and Raymond 2021). Importantly, this research found that a vision of wildlife gardening as caring for nature and community, a practice that is feasible and meaningful for residents and connected to their everyday lives and the places they live in, underpinned program development and multiplication (Mumaw and Mata, in press).

## The way forward: Lessons from and implications for Australian cities

The COVID-19 pandemic has shone a spotlight on human-nature interactions, inequities of access and other issues of environmental justice (Morse et al. 2020; Diffenbaugh et al. 2020) in cities of all sizes. The pandemic has led to citizens in cities across the world increasing their usage of parks and re-assessing their connection to nature, prioritising access as part of limited freedoms in city lockdowns. Spending time in nature has contributed to stress relief during the social and individual disruptions of the pandemic (Egerer et al. 2022). As the recent Regen Melbourne (2021) report concludes: “it’s not just about building green compromises to existing city areas, but to shift what development looks like in a city, where we put nature back into the heart of how we plan for tomorrow’s urban environment”. Inequity of access to green space has been particularly highlighted during the pandemic, when limitations on travel have restricted urban residents to their local areas; residents without access to local green spaces have reported increased feelings of deprivation (Ugolini et al. 2021). The studies drawing data and observations during the pandemic on how important urban green spaces are as places for human health improvement and sustainability amplify the messages (and key insights from numerous studies before the pandemic) about the multiple benefits deriving from urban nature: for nature/biodiversity, for climate resilience and overall for human and planetary health and pointing to the urban inequalities that manifest via unequal access, uneven or non-intersectional urban design of urban green and blue spaces in cities (Jasinski 2022; Yap et al. 2022). Focussing on provision of access to urban green spaces and opportunities for gardening can be part of a ‘public health strategy, readily accessible to boost societal resilience to disturbances’ (Egerer et al. 2022, p. 1), but a consistent focus on providing opportunities for older citizens and those in lower socioeconomic areas is needed (Levinger et al. 2021). The Covid19 pandemic also revealed the importance of both private green spaces and publicly managed and owned urban ecosystems for dealing with mental distress and improving general well-being of urban citizens (Basu et al. 2021; Wortzel et al. 2021; Noszczyk et al. 2022). Likewise, presenting green infrastructure projects as part of economic stimulus projects, as seen in early responses Covid-19—as seen in an assessment of 100 city response in the first half of 2020, such as the City of Melbourne’s biodiversity planting. The lesson here is prioritising green infrastructure as a job creation exercise, with multiple other social and environmental benefits (Hadfield et al. 2021). Holistic responses, in planning equitable networks of urban green spaces, must also further integrate Aboriginal environmental philosophy and knowledge into mainstream sustainable development practices. We know that the current generation is more likely to live in cities, and will need to connect with nature and with the world’s oldest continuous culture (Miller 2021).

It has been almost 10 years since cities were placed front and centre by the Secretariat on the Convention of Biological Diversity (2012). Since then, nature-based solutions have entered the New Urban Agenda and there have been growing calls to use cities as learning spaces and agents of change to spearhead a transformative agenda in the global biodiversity framework. We add our calls to theirs and here have outlined three key pathways: establish evidence-based planning for nature in cities; strengthen collaborative planning and inclusive governance for nature-based solutions and empower communities to innovate with nature. We have illustrated these with practical, innovative examples. Whilst we focus on Australian cities, the lessons and pathways are broadly applicably globally. Meetings of the Convention on Biological Diversity and UN Convention on Climate Change in 2021 will shape the global responses to biodiversity loss and climate change for the current decade. Cities and residents of cities play a critical role in the response but transformations in the way cities and their residents consider and embrace nature, such as outlined in this article, are urgently needed.
